# A Review of NDT Methods for Wheel Burn Detection on Rails

**DOI:** 10.3390/s23115240

**Published:** 2023-05-31

**Authors:** Yanbo Zhang, Xiubo Liu, Longhui Xiong, Zhuo Chen, Jianmei Wei

**Affiliations:** 1 Postgraduate Department, China Academy of Railway Sciences, Beijing 100081, China; yanbo_z@163.com (Y.Z.); 19952749989@163.com (J.W.); 2 Infrastructure Inspection Research Institute, China Academy of Railway Sciences Corporation Limited, Beijing 100081, China; xionglonghui@rails.cn (L.X.); cz970429@163.com (Z.C.)

**Keywords:** rail defect, wheel burn, NDT, white etching layer, mechanism of formation

## Abstract

Wheel burn can affect the wheel–rail contact state and ride quality. With long-term operation, it can cause rail head spalling or transverse cracking, which will lead to rail breakage. By analyzing the relevant literature on wheel burn, this paper reviews the characteristics, mechanism of formation, crack extension, and NDT methods of wheel burn. The results are as follows: Thermal-induced, plastic-deformation-induced, and thermomechanical-induced mechanisms have been proposed by researchers; among them, the thermomechanical-induced wheel burn mechanism is more probable and convincing. Initially, the wheel burns appear as an elliptical or strip-shaped white etching layer with or without deformation on the running surface of the rails. In the latter stages of development, this may cause cracks, spalling, etc. Magnetic Flux Leakage Testing, Magnetic Barkhausen Noise Testing, Eddy Current Testing, Acoustic Emission Testing, and Infrared Thermography Testing can identify the white etching layer, and surface and near-surface cracks. Automatic Visual Testing can detect the white etching layer, surface cracks, spalling, and indentation, but cannot detect the depth of rail defects. Axle Box Acceleration Measurement can be used to detect severe wheel burn with deformation.

## 1. Introduction

Rails, as the key component of the railway system, are subjected to intense bending, shear, contact, and thermal and residual stresses during operation [1]. These complex loading conditions may cause formation and deterioration of different defects which mainly originate from manufacturing, inappropriate handling, and rolling contact fatigue or corrosion [2]. Among these defects, wheel burn has lately received much attention.

In recent years, wheel burn has been detected on many high-speed railway lines in China at the early stage of operation [3,4,5,6,7,8]. Wheel burn accounts for around 36% of the total defects on 17 high-speed railway lines in China [9]. Wheel burn can affect the wheel–rail contact state and ride quality, and under long-term operation, it may cause cracking, spalling, or even rail breakage.

Research on wheel burn can be divided into the macro-scale and micro-scale. Macro-scale research involves the study of characteristics and causes of wheel burn, while at the micro-scale, the mechanism of formation and crack extension of the white etching layer (WEL) formed by wheel burn have captured the attention of some scholars.

### 1.1. The Characteristics and Causes of Wheel Burn

**Figure 1:**
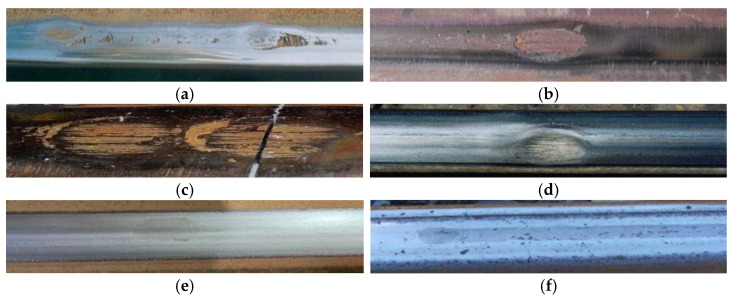
Macroscopic appearance of wheel burn [10,11]. (**a**) Repeated wheel burn caused by locomotive running; (**b**) Rail surface spalling caused by wheel burn; (**c**) Transverse breakage caused by wheel burn; (**d**) Oval-shaped wheel burn caused by locomotive slipping; (**e**) Oval-shaped WEL caused by wheel burn; (**f**) Strip-shaped WEL caused by wheel burn.

In terms of the macro-scale, the characteristics and causes of wheel burn have been described according to many standards. In UIC712, wheel burn is classified as either single wheel burn or repeated wheel burn [10]. Single wheel burn is identified as an elliptical white self-quenched layer, while repeated wheel burns are identified as a combination of single wheel burns, which are characterized by a wavy rail surface (see Figure 1a). They are caused by wheel-slipping (when the length traveled by a wheel during one rotation cycle is less than its circumference), breaking (also called wheel-skidding, where the length traveled by a wheel during one rotation cycle is larger than its circumference), or wheel-slipping while moving. They mostly occur at signals and railway stations. Under long-term operation, horizontal and transverse cracks developed from wheel burn may cause rail head spalling or rail breakage. Australian Railway [11] claims that excessive slope, improper driver operation, insufficient traction, and decreased friction caused by rail surface contamination will lead to locomotive slipping during the starting and operating stages, resulting in wheel burns. Mild wheel burn may gradually develop into spalling (see Figure 1b), while severe wheel burn may cause transverse cracks and lead to rail fracture (see Figure 1c). The US Railway [12] claims that thermal cracks formed by the consecutive increase in rail surface temperature due to locomotive slipping and then rapid decreases when the train leaves, can cause the transverse fracture of the rail. Typically, multiple wheel burns will appear within a relatively short distance. According to Chinese standards [13], wheel burn is caused by wheel-slipping during locomotive start-up. Wheel burns are elliptical, are distributed symmetrically on both rails, and have spacing equal to the locomotive wheelbase. Continuous longitudinal wheel burns or repeated wheel burns may lead to spalling or transverse breakage.

Wheel burn usually appears as an oval-shaped or strip-shaped white self-quenching layer, with approximately 0.3–2.5 mm depth, 15 mm–10 m length, and 15–30 mm width, located on the running surface of the rail [14]. It is usually distributed near home and departure signals, long ramps, and small-radius curves [9,15]. Based on its morphological and distribution characteristics, it can be deduced whether wheel burn is produced by wheel-slipping or wheel-skidding [6,9]. When a vehicle starts or runs, insufficient traction force will cause wheel-slipping, by which oval-shaped WEL will be produced. In more serious cases, rail tread depression will be created immediately, and a hump can be observed at the edge of the depression because of the plastic flow (see Figure 1d). Likewise, when the vehicle brakes, wheel-skidding will cause the production of oval-shaped or strip-shaped WELs (see Figure 1e,f). These WELs will develop into rail head spalling, transverse cracks, and other rail defects under periodic wheel–rail contact loading [16,17].

### 1.2. The Mechanism of Formation and Crack Extension of the White Etching Layer

The formation mechanism, microstructure, and crack propagation of WEL have been studied at the micro-scale by many scholars; however, the results are still a matter of controversy [18,19,20]. The conception of the white layer was initially presented by Stead [21] in 1912 after the discovery of the white layer on steel wire rope. Since then, the WEL has been found in many different situations on both ferrous and non-ferrous materials [22]. Based on earlier research, WEL, which is considered to be a form of martensite, is formed by a sequential process during wheel-skidding, in which temperature rises beyond the austenitization temperature, followed by rapid cooling (or quenching) [23,24,25,26,27]. By investigating the microstructure of WEL from rails, retained austenite has been found by multiple researchers, which provides strong proof of a thermal-induced formation mechanism [28,29,30]. Except for the heat source generated by wheel–rail contact, electrical arcing caused by current leakage is a new potential heat source of the thermal-induced formation mechanism of WEL [31,32]. However, this thermo-induced theory is objected to by other researchers [33,34,35,36,37,38], who believe that the austenitization temperature of the wheel–rail contact zone is not reachable under normal rolling conditions. Instead, WEL is considered to be a nanocrystalline Fe-C alloy with a high density of dislocations and occasional twins, which is supersaturated by carbon because of severe plastic deformation. Furthermore, a 2GPa contact pressure loading and a high-pressure torsion experiment were applied to pearlitic rail steel, and WEL was produced under this condition of pure severe plastic deformation. This verified the plastic-deformation-induced mechanism [32]. Recently, another verification experiment to reproduce WEL under pure plastic deformation conditions has been conducted by Pierrick et al. on a circular rail test bench [39].

**Figure 2:**
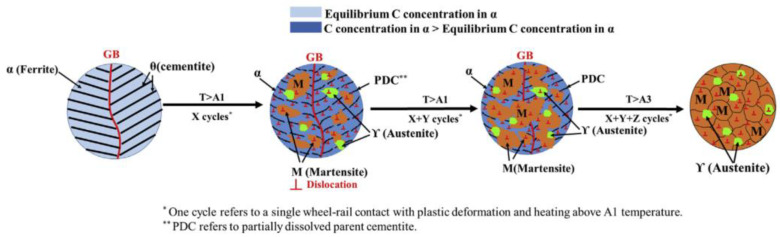
Schematic drawing of thermomechanical mechanism of WEL formation [29].

Based on these two formation mechanisms, contradiction is quite easy to find. If severe plastic deformation is the only mechanism, the sharp phase transition process is unable to be explained. In addition, if thermal phase transformation is the only mechanism, WEL will be produced within one cycle of loading, which cannot explain why WEL is loading-history-dependent [40]. Hence, WEL is believed to be formed by a combination of these two mechanisms [25,41,42,43,44,45,46,47]; the thermomechanical mechanism of WEL formation is illustrated in Figure 2. At the initial cycling stage, pearlitic starts to transform to austenite when the temperature rises above A1, and some of the formed austenite is transformed to martensite during the cooling process. At the same time, cementite dissolves due to plastic deformation and temperature rise, and a nanocrystalline structure is produced. After that, martensite and austenite grow gradually during cyclic loading, and a large austenite area is formed. At the end of the cooling stage, some part of austenite has been transformed into martensite, while some of it remains as austenite. However, full austenitization is needed to completely dissolve cementite and form martensite and austenite, which requires more cycles with temperatures beyond A3 [29].

**Figure 3:**
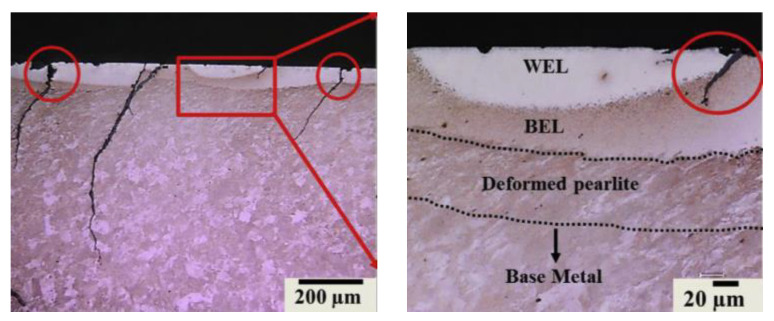
Optical micrograph of the rail cross-section with WEL, BEL, and cracks [29].

To summarize, the microstructures of WEL are considered to be: (1) martensite [48,49]; (2) nanocrystalline Fe-C alloy [36]; (3) martensite and retained austenite [24,28]; (4) deformed pearlite, nanocrystalline martensite, austenite, and cementite [29,43]. Interestingly, a new tribologically transformed structure named brown etching layer (BEL) has been found near WEL and has been studied in recent works (See Figure 3) [29,40,50,51,52]. Usually, BEL coexists with WEL, forming an internal sandwich structure. BEL is considered to be thermal-induced or thermomechanical-induced, but the mechanism of its formation is not clear yet.

**Figure 4:**
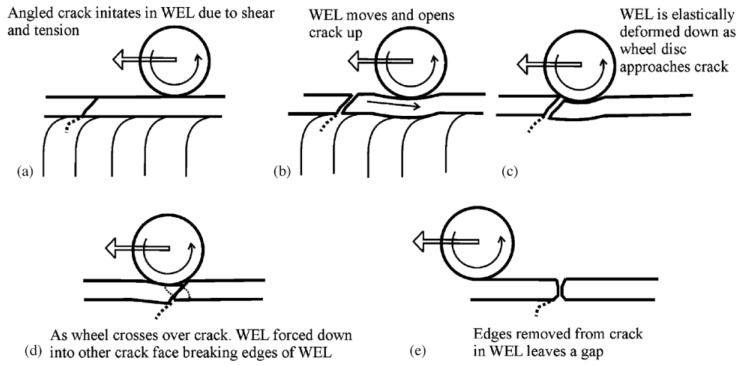
Schematic of crack development as wheel rolls over [58].

Because WEL is hard (up to 1200 HV) and brittle, small cracks usually initiate from here and propagate to generate other rail defects [53,54]. Cracks usually appear at the edge and in the middle of the WEL area. After crack initiation, cracks will penetrate from the WEL area to the parent rail matrix, and grow into the rail matrix [55,56,57]. Figure 4 [58] explains the propagation mechanism of microcracks generated by the white etching layer microstructure under the loading of moving wheels. In the process of rolling forward, the wheelset acts on the rail before and after the microcrack, making the crack open and forcing different fracture surfaces of the white etching layer to contact, which causes the crack edge to wear, and makes the crack grow and expand continuously, thus leading to other defects such as spalling. Based on the theory of Liu et al. [17], the WEL produced by wheel burn of starting or braking of the high-speed train is thinner (less than 0.2 mm), and is more likely to develop into cracks, while the WEL formed by a heavy axle load locomotive is thicker (greater than 0.5 mm), and is more likely to develop into spalling.

**Figure 5:**
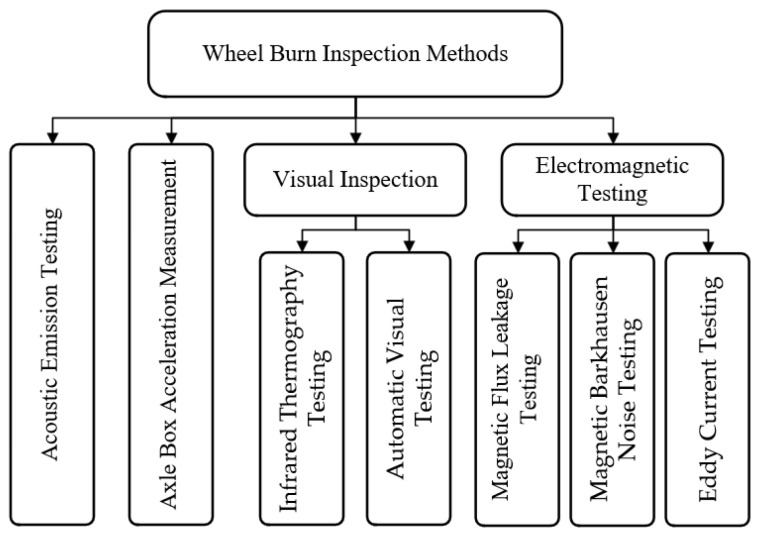
Classification of the NDT methods for wheel burn detection.

To avoid catastrophic outcomes caused by wheel burns, accurate identification at the early stage of wheel burns is necessary. Currently, optical microscope and hardness tests are used to identify and characterize WEL, which are effective but destructive, expensive, and time-consuming [59]. NDT methods are better choices to overcome these weaknesses, but there is no such widely researched method commonly used for WEL detection. At present, the main methods of rail surface defect detection are: Magnetic Flux Leakage (MFL) Testing, Magnetic Barkhausen Noise (MBN) Testing, Eddy Current (EC) Testing, Acoustic Emission (AE) Testing, Inferred Thermography (IRT) Testing, Automatic Visual (AV) Testing, Ultrasonic Testing (UT), and Axle Box Acceleration (ABA) Measurement. Although UT can detect some wheel burns, its sensitivity to surface and near-surface defects is low and it is mostly used for internal damage detection [60,61]. Therefore, this paper will discuss the detection methods listed above, except for ultrasonic testing. The classification diagram of the NDT methods for wheel burn detection is shown in Figure 5.

This paper focuses on methods for detecting rail surface and near-surface defects, which are suitable for wheel burn detection. The first section demonstrates the characteristics, causes of wheel burn, and the mechanisms of WEL formation and crack extension. The second, third, fourth, and fifth section discuss electromagnetic testing, AE testing, visual inspection, and ABA measurement, respectively, including principles and state-of-the-art applications. The sixth section summarizes the findings and perspectives.

## 2. Electromagnetic Testing

### 2.1. Magnetic Flux Leakage Testing

**Figure 6:**
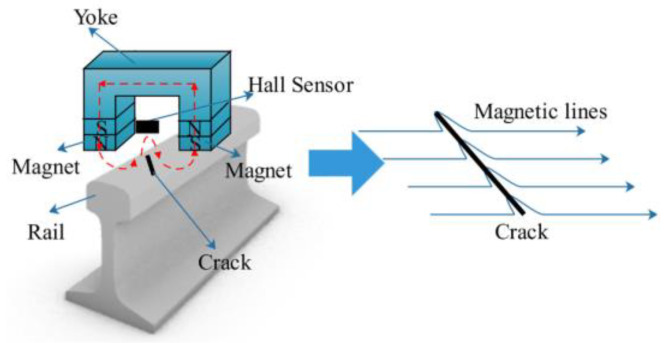
The principle of MFL testing of rail [64].

As a mature electromagnetic NDT method, MFL testing has already been used for inspecting storage containers, rails, pipelines, etc. When a magnetic material is magnetized, defects on its surface cause changes in magnetic permeability, which shifts the magnetic flux and alters the orientation of the magnetic induction line. This results in magnetic flux leaking to the surface of material, bypassing defects in the atmosphere, and then re-entering the material, creating a leakage magnetic field. Magnetic field signals from the surface will be collected by magnetic sensors to identify rail defects [62,63,64]. The principle of MFL testing is shown in Figure 6.

MFL testing is suitable for detecting surface and near-surface cracks, and most researchers are focusing on how to improve the speed, efficiency, and resolution of crack detection [65,66,67,68]. Based on the principle of MFL testing, the magnetic flux leakage is caused by changes in magnetic permeability because of the existence of defects [69]. Formation of WEL may experience phase transformation, grain refinement, dislocation concentration, plastic deformation, etc. Most changes in microstructure will result in changes of magnetic permeability of the component [70], which will cause the leakage of magnetic flux and be captured by the magnetic sensor. Therefore, MFL testing is a possible detection method for WEL.

Under periodic wheel–rail interaction, cracks will initiate and grow from the WEL. In order to determine the size of near-surface and far-surface cracks, Ali et al. [71] proposed a pulsed MFL technique for crack detection and characterization, and its feasibility was shown using simulations and experiments. By using magneto-optical sensors, Tehranchi et al. [72] investigated the relationship between the sensor signal and the length of the cracks, which has great potential to obtain high-resolution magnetic imaging of small cracks.

In addition, Yong et al. [73] proposed a 3D MFL testing method to achieve arbitrary defect detection on rail tracks, and the results gave comprehensive information on the position and shape of defects.

**Figure 7:**
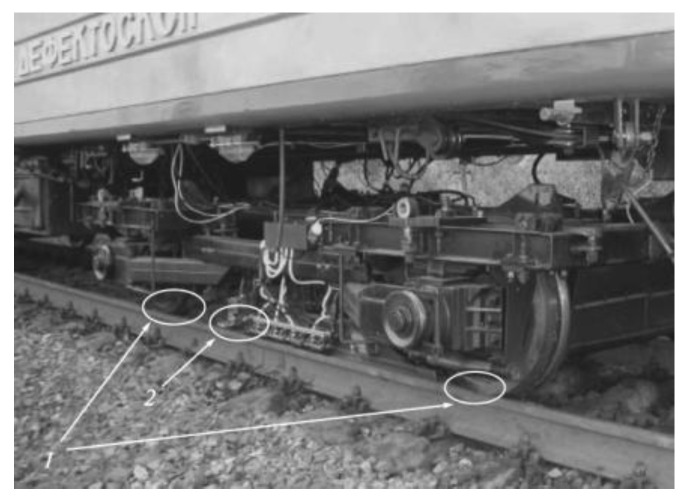
Russian rail flaw detection vehicle AVIKON-03 with magnetizing system: (1) the maximum magnetic induction region, and (2) magnetic sensor [80].

As early as the late 1920s, Sperry Company had developed the induction method for rail inspection, which provided a solution for crack detection [74]. The team from Nanjing University of Aeronautics and Astronautics (NUAA) carried out research on different aspects of MFL testing, inspection speed, lift-up distance, and other influencing factors, as well as improving the detection probe [65,75,76,77,78]. In China, an MFL testing system has been installed in the GTC-80X rail defect detection vehicle, and on-board tests are completed with a running speed of 40 km/h [79]. Antipov et al. [80] investigated the effectiveness of the MFL system installed on the Russian rail defect detection vehicle AVIKON-03 (See Figure 7) under the inspection speed of 80 km/h, and adjusted the interpole distance accordingly.

### 2.2. Magnetic Barkhausen Noise Testing

**Figure 8:**
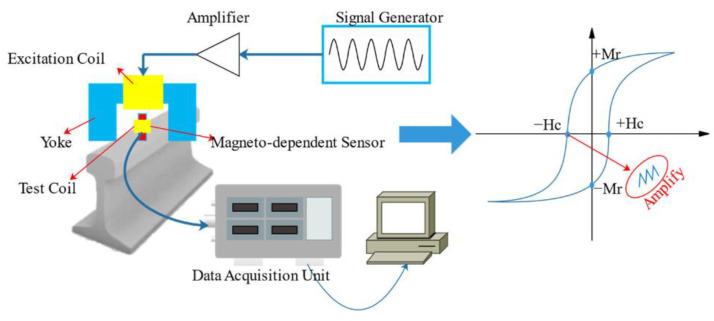
The principle of MBN testing on rails [64].

The MBN signal is a pulsed current signal produced by the movement and deflection of magnetic domains (Bloch wall motion) within a ferromagnetic material under an alternating magnetic field, which is sensitive to changes in stress condition and microstructure of the material [81]. The microstructure of the rail matrix can be described by carbide precipitation [82], dislocation density [83], grain size, or non-ferromagnetic phases [84]. These microstructure changes may occur during the formation process of WEL, so MBN inspection is a feasible method to achieve WEL detection. The principle of MBN testing is shown in Figure 8.

**Figure 9:**
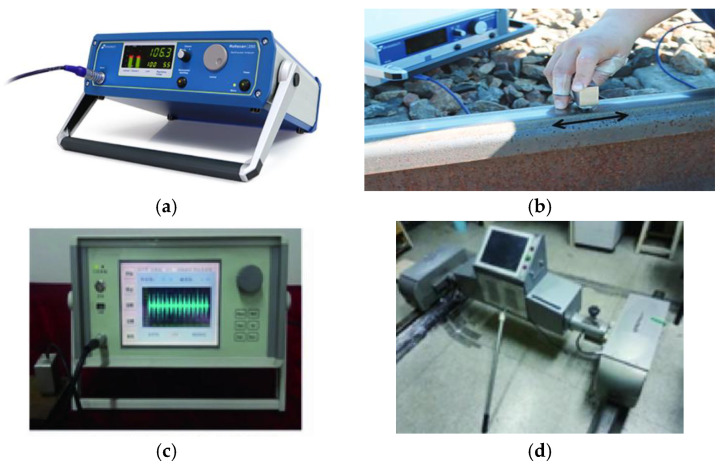
MBN testing devices [81,90]. (**a**) Rollscan 250 from Stresstech (Finland); (**b**) MBN measurement at rail surface with Rollscan 250; (**c**) MBN stress detector from NUAA; (**d**) Hand-pushed rail stress and crack inspection device from NUAA.

A simple way to identify WEL is to compare hardness between WEL and the rail matrix; MBN inspection is a feasible way to achieve this hardness measurement [85]. In studies by Neslušan et al. [86,87], an evidently lower MBN signal was found in the thick WEL, which is due to: 1. Carbon-supersaturated martensite has a high degree of lattice tetragonality and high hardness; 2. Retained austenite exists in WEL and hinders the Bloch wall motion; 3. Oxides (FeO and Fe_2_O_3_) and carbides pin the Bloch wall motion; 4. Microcracks do not produce MBN emissions. Similarly, Jiang et al. [88] built a non-destructive testing system based on MBN technology, and investigated how MBN signals were affected by prefabricated WEL on U75V rails. Compared to the rail matrix, the appearance of WEL causes a rapid decrease in MBN signal. Furthermore, with an increase in WEL thickness, the strength of the MBN signal becomes weaker. From the perspective of quantitative analysis, the peak-to-peak value and RMS value of the MBN signal show a good linear negative correlation with the thickness and area of WEL, which means the appearance and thickness of WEL can be described by the MBN signal. However, MBN signals are temperature-related, so temperature changes need to be considered when conducting the MBN inspection. In order to eliminate the temperature effect, Wang et al. [89] found that the MBN signal has a monotonic relationship with temperature, and so created a back propagation (BP) neural network model to obtain an accurate stress value. At present, some MBN equipment has already been used for detecting rail surface condition, such as the Rollscan 250 from Stresstech Company and the rail stress and crack inspection trolley from NUAA (See Figure 9).

### 2.3. Eddy Current Testing

**Figure 10:**
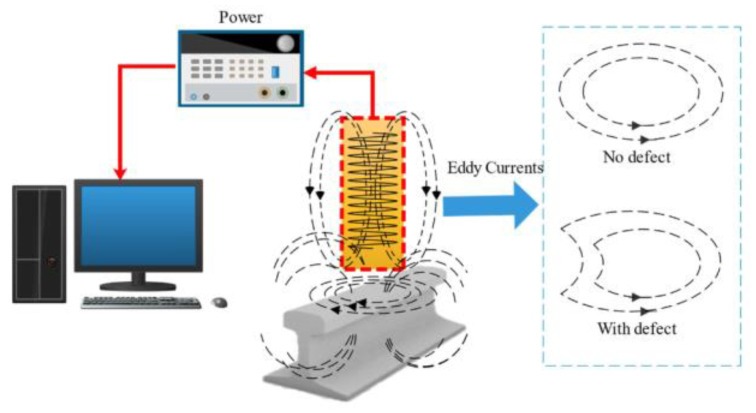
The principle of EC testing of rail [64].

Due to the principle of electromagnetic induction, alternating current in a coil sensor creates a changing magnetic field, which will produce an eddy current on the rail surface. The eddy current will produce a new magnetic field, the direction of which is opposite to that of the initial magnetic field. Thus, when defects exist, the fluctuation in difference between the two magnetic fields will cause variation in the impedance and electromotive force [91,92]. Eddy current detection is appropriate for rail surface and near-surface flaw detection, since it is more sensitive to small cracks. The skin effect causes the intensity of eddy currents to diminish from the surface of the specimen downwards, and so detection depth is related to excitation frequency. The principle of EC testing is shown in Figure 10.

**Figure 11:**
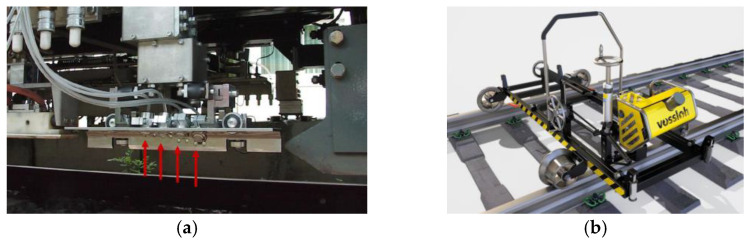
EC testing devices [94,99]. (**a**) German rail inspection vehicle with eddy current sensors installed (red arrows); (**b**) Rail grinding system with integrated EC inspection module from Vossloh.

The different microstructures of WEL cause different electrical conductivity compared to the rail matrix, which can be captured by a coil sensor to identify WEL at an early stage. By using a four-channel portable eddy current detector, Xiong et al. [93] conducted on-site testing of 10 wheel burns. By extracting the characteristic voltage signals of wheel burns, damage levels are classified based on the length and width of wheel burns. Therefore, wheel burn (or WEL) can not only can be detected by the EC testing method, but can also be analyzed quantitatively. As shown in Figure 11 [94], Thomas et al. studied the ability of the ultrasonic and eddy current inspection device installed on rail inspection vehicles used by a German railway company, and the results showed that eddy current inspection equipment could determine the location and size of rail surface defects, such as wheel burns, rail head cracks, and squats. It should be mentioned that eddy current is sensitive to rail surface roughness, so rusting and corrosion may affect detection accuracy [95].

Huang [96] conducted experiments on the quantitative assessment of eddy current detection of cracks on the rail surface; due to the skin effect, detection results are not accurate if the depth is greater than 5 mm. By conducting simulation and experiment analysis, a new technique for non-destructive testing based on velocity-induced eddy currents was proposed and validated by Ramos et al. [97]. As the sensitivity of this method increases with speed, it shows a great advantage for on-board inspection of rail. Liu et al. [98] proposed an electromagnetic tomography (EMT) technique to achieve crack detection on the rail surface. They used tomographic methods to measure the alternating magnetic signals from cracks and reconstruct the distribution of the cracks, which can obtain the shape and location of cracks.

For the EC testing device, the Vossloh company developed a rail grinding system with an integrated EC inspection module, which can detect rolling contact fatigue defects after rail grinding [99]. By using an eddy current, Huang et al. [100] developed a rail surface detection system that is applicable for both switches and mainline rails; Hu et al. [101] developed an eddy current detection method and device that can quantitatively and accurately detect small cracks on the rail surface; Park et al. [102] designed a hand-pushed 16-channel eddy current detection device, which is more accurate at measuring the length and width of rail surface damage.

## 3. Acoustic Emission Testing

**Figure 12:**
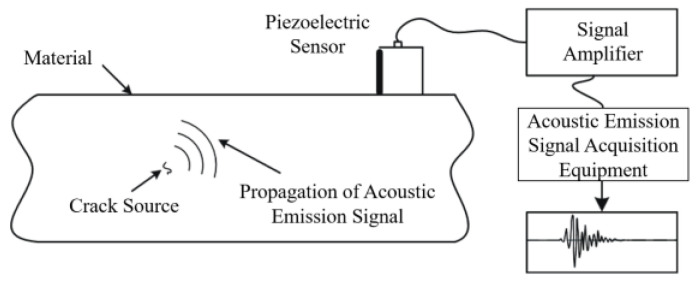
The principle of AE testing [64].

**Figure 13:**
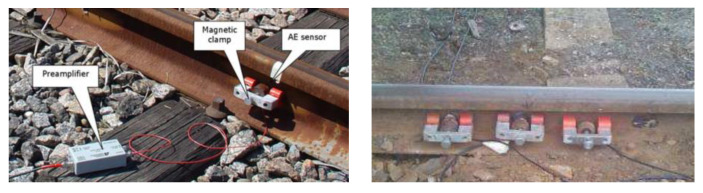
Installation of AE sensor on rail [107,111].

The rapid release of energy from an object or substance causes a transient elastic wave to be created, which is known as acoustic emission [103,104]. Usually, plastic deformation and cracks of materials are the main sources of acoustic emission, as well as phase changes, corrosion, friction, and magnetization [105]. The elastic waves generated by AE sources are conducted to the surface, causing vibration of surface particles, which is captured and converted into an electrical signal by a piezoelectric transducer, processed by an amplifier, and collected by the instrument. The frequency range of AE testing is typically 20 kHz to 1 MHz [106]. The principle of AE testing is shown in Figure 12. In practice, the sensor is usually fixed to the flat surface of the rail web through clamps, and the couplant is needed [107]. The onsite installation is shown in Figure 13.

AE testing is frequently used for monitoring rail cracks and wheel defects. Muravev et al. [108] have analyzed the stress–strain conditions of rails when trains are passing, taking into account the effects of mechanical and electromagnetic noise on detection, and have theoretically demonstrated that cracks in rails can be detected using acoustic emission methods. Bianchi et al. [109] researched the development of rail head fatigue defects by using individual AE sensors, and the wavelet packet approach was used to extract the signal character. To simulate high-speed situations, Xin et al. [110] constructed a wheel–rail platform that can accelerate up to 177 km/h, and by using the time–Shannon entropy method, defect signals can be detected at 124 km/h. BOLLAS et al. [111] analyzed AE signals from rail-installed AE sensors, as shown in Figure 13, and captured different characters of AE signals of damaged and non-damaged wheels, which demonstrates the potential of AE testing for wheel defect diagnosis.

As for WEL, increased brittleness causes decreased damping, which will result in a larger amplitude of vibrations [59]. Based on the research of Guo et al. [112], the existence of WEL increases the amplitude and frequency of the AE signal, which provides fundamental information for WEL detection. Surface roughness is another important factor that can affect the AE signal, so the noise generated by rough surfaces needs to be eliminated. From the perspective of microstructure, by analyzing the AE characteristics of the martensitic transformation process, Jiang et al. [113,114,115] propose a new dynamic monitoring method for martensitic transformation based on AE, and obtained accurate laws of martensitic transformation, which provides a theoretical basis for WEL identification.

## 4. Visual Inspection

### 4.1. Infrared Thermography Inspection

Thermal imaging detection, which can also be referred to as IRT inspection, can be classified into active and passive types depending on how the temperature change is caused. Active IRT inspection uses external thermal input to cause the object to lose thermal equilibrium and to detect infrared before it reaches equilibrium internally, while passive IRT inspection uses the heat exchange between the object and its surroundings to achieve infrared detection [116].

Because rails cannot heat themselves, rail defect detection is generally carried out using active IRT inspection. Depending on the heating method, active IRT inspection can be divided into pulsed thermography, step heating thermography, lock-in thermography, laser thermography, and ultrasonic thermography [116].

In pulsed thermography, a high-frequency alternating current is brought close to the rail surface, which induces eddy currents with different densities because of defects. Based on Joule’s law, eddy currents are further transformed into heat, creating high- and low-temperature regions within the material and inducing temperature variation at the material surface through heat conduction. The temperature anomalies are recorded by a thermal imaging camera and processed to extract the defect information [117,118]. This testing method is used more for rail crack detection. Peng et al. [119] used eddy current pulsed thermography to quantitatively investigate the characteristics of closed cracks and natural cracks produced by rolling contact fatigue, and established a model of closed cracks with rust as a filler. The results demonstrated that the higher temperature at the crack tip is caused by a higher eddy current density, and that the maximum temperature at the crack tip rises with increasing crack length. Bai Jie et al. [120] investigated the crack closure effect caused by the filling of a third substance by using pulsed thermography. Feng et al. [121] researched the progress of squat crack growth by using eddy current pulse thermography and verified the feasibility of eddy current pulse thermography detection for squat identification.

**Figure 14:**
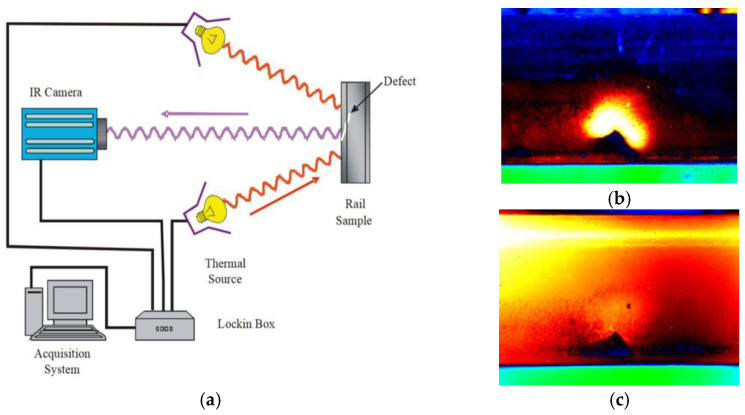
Detection of squats by lock-in thermography [123]. (**a**) Schematic of lock-in thermography experiment setup; (**b**) Phase angle image of squat; (**c**) Temperature image of squat.

While pulsed thermography is not very sensitive to small-sized or fatigue defects, the phase information of lock-in thermography effectively reduces the impact of noise and improves the signal-to-noise ratio of the temperature signal, so the location and shape of defects can be accurately determined by extraction of phase information [122]. As shown in Figure 14a, by using heat lamps as a heat source, Daren et al. [123] successively identify the appearance and location of squats, but their measurement of squat depth was not accurate based on angle images. Likewise, Peng et al. [124] also investigated the reliability of lock-in thermography for squat identification. The results showed that lock-in thermography can accurately identify squats of different sizes and depths with higher sensitivity than ultrasonic testing. In addition, because the amplitude of heat input and convection loss are not able to change the phase response of the rail surface, phase images can only be affected by material properties and defect geometry. Lv [125] conducted crack extension experiments on the head, web, and bottom of U71Mn rails, and proved the feasibility of this method by comparing the results with the traditional compliance method and visual method.

In laser thermography, the laser is used as a heat source to heat the surface of the defect, and an infrared imaging camera is used to measure the thermal dispersion during the heating process, thus the characterization of the defect is captured [126]. Based on the research of Giuseppe [127], laser thermography was used to distinguish the ferritic and martensitic structure in boron steel and found a difference in the thermal diffusivity between the two of 35%. There are thermal diffusivity differences among different phases of steel, which means the martensite phase of WEL can be identified by using laser thermography.

### 4.2. Automatic Visual Testing

Recently, AV testing has been widely used for track inspection. AV testing mainly uses cameras to obtain image signals, and then image processing systems are used to convert image signals into digital signals so that computers can make reasonable judgments instead of human eyes. However, similar to human eyes, machine vision can only detect rail surface defects, and internal cracks or near-surface defects are not detectable. At present, some developed countries have established some industry systems and standards, which have been broadly used in the railway industry, among which the representative ones are [128]:

1. The RSIS system developed by ENSCO in the United States uses the line scanning imaging method to collect and record continuous high-resolution track surface images from moving vehicles, and can successfully identify spalling, cracks, squats, wheel burns, etc.;
2. The V-CUBE rail vision inspection system developed by the MERMEC Company in Italy can obtain images and inspection data of up to 50 types of defects of rail surfaces, fasteners, sleepers, and ballast beds at a speed of 200 km/h;
3. The RailCheck system developed by the bvSys Company in Germany uses an industrial linear scanning camera and high-powered LED light to inspect rail surfaces, fasteners, sleepers, turnouts, and other structures at a speed of 200 km/h.

**Figure 15:**
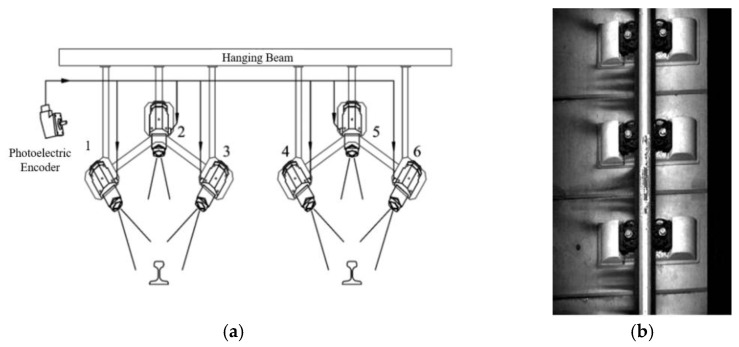
A vehicle-mounted track inspection system developed by China Academy of Railway Science [129,130,131]. (**a**) The principle of track inspection (1–6 are line scan cameras); (**b**) Wheel burn detected by track inspection system.

A vehicle-mounted track inspection system has been developed by China Academy of Railway Science [129,130,131], which uses six linear CCD cameras to scan the full track (See Figure 15). Principal component analysis and linear discriminant analysis are used to build a machine learning model to realize automatic identification of wheel burns and other defects. The system has achieved a 95% detection rate of wheel burns with a 160 km/h inspection speed. Ren et al. [132] proposed a wheel burn identification algorithm, which mainly includes four processes: rail region extraction, gray contrast image generation, binarization based on the maximum entropy principle, and wheel burn determination. They achieved a detection rate of 90.7%. Based on this research, Zhao et al. [133] proposed a wheel burn detection algorithm based on spatial filtering. In this algorithm, the mean gray value of the original image is used to fill the defect area, which enhances the contrast between the wheel burn area and the background area in the gray image, therefore improving the wheel burn detection rate.

**Figure 16:**
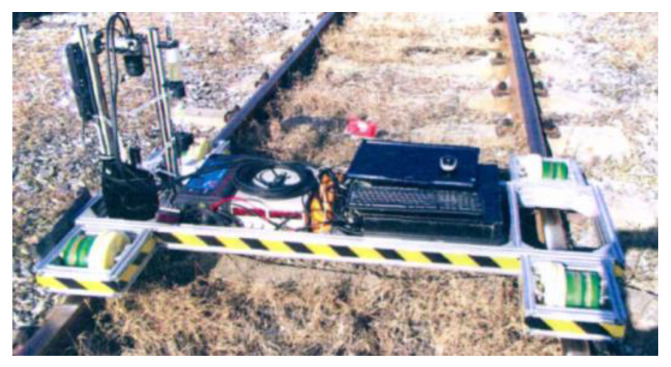
Track inspection trolley [128].

**Figure 17:**
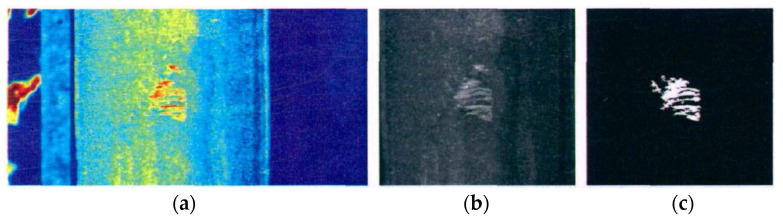
Photograph of wheel burn detection [128]. (**a**) Target image; (**b**) Detection region; (**c**) Wheel burn.

Ding [128] designed an experimental portable rail surface detection trolley with a 4K linear camera and extracted the wheel burn features by using the maximally stable extremal regions method (MSER), realizing the automatic identification of wheel burn. The track inspection trolley and identification process are shown in Figure 16 and Figure 17, respectively. Based on the principle of blind source separation, Li et al. [134] proposed a sparseness representation model of defect images and realized the recognition of wheel burn.

With the aid of the deep learning technique and the Cuda parallel computing architecture, Santur et al. [135,136] were able to classify rail surface defect images captured by 3D laser cameras. With 99% detection accuracy, this technique can identify rail surface defects in real-time at speeds of up to 144 km/h. Shang et al. [137] proposed a sequential processing method that uses Canny edge detection to locate the rail and uses a convolutional neural network (CNN) to classify and identify the rail images with a 92.08% detection accuracy. A rail surface detection method was proposed by Tangbo et al. [138], which improves the detection rate of minor defects on the rail surface by building a lightweight and quick-response improved YOLOv4 model.

## 5. Axle Box Acceleration Measurement

The axle box and wheelset are rigidly connected, and the short-wave irregularity of the rail excites the vibration of the wheelset, which will transmit to the axle box directly, so the ABA can reflect the disturbance caused by rail defects to a certain extent, which means some rail defects can be identified by using ABA [139]. For mild wheel burn with phase transformation only, usually, no excitations can be detected by the ABA sensor. However, for severe wheel burn with plastic deformation or spalling developed from wheel burn, obvious excitation signals can be detected by ABA detection.

**Figure 18:**
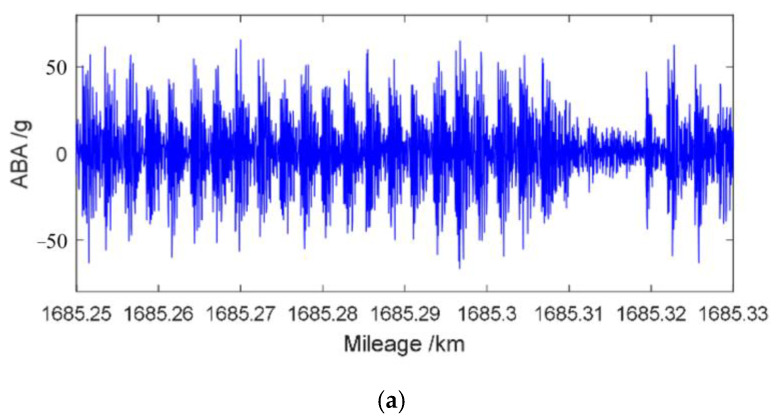

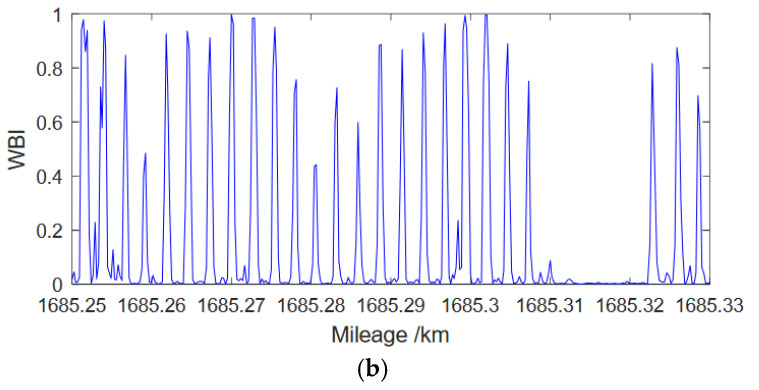
Original ABA signal and WBI signal of continuous and equidistant wheel burn [145]. (**a**) Original ABA signal; (**b**) WBI signal.

Much research has been carried out on evaluating rail state by using ABA, among which Molodova et al. [140] used wavelet power spectrum to classify squats of different severity based on the frequency domain response of the ABA signal, and could differentiate the severity of squats based on frequency magnitude. The detection rate of minor squats was 78%, while the detection rate of moderate and severe squats was 100%. Zili et al. [141] proposed three improved methods for detecting minor squats based on ABA. The first method is to use longitudinal ABA to improve the sensitivity of squat detection. The second method is to use multi-sensors, adopt noise reduction technology, and repeat measurement. The third method is to reduce the interference of wheel defects using signal processing. Cao et al. [142] used the Hilbert–Huang transform method to perform time–frequency analysis of ABA, and realized localization of short-wave irregularity. By using the maximum power spectral density, Jamshidi et al. [143] evaluated the length of squat from ABA signals and established a robust model to predict the growth of squat. Liu et al. [144] proposed a track impact index method based on vertical ABA, which reduces the influence of random factors such as rail joint welds and turnouts on the results, and achieves the identification of short-wave irregularity defects. XU et al. [145] proposed a wheel burn index (WBI) to identify continuous and equidistant wheel burn, which uses a demodulated envelope signal to replace the original signal, so an energy signal with low-frequency is extracted to achieve efficient wheel burn diagnosis. The original ABA signal and WBI signal of continuous and equidistant wheel burn can be seen in Figure 18.

## 6. Summary and Perspective

In summary, the characteristics, mechanism of formation, crack extension, and detection methods of wheel burn are reviewed, and the main findings and conclusions are summarized below:

1. Initially, the wheel burns appear as an elliptical or strip-shaped white etching layer with or without deformation on the running surface of the rails. In the latter stages of development, they may cause cracks, spalling, etc. Three main mechanisms of WEL formation have been proposed by scholars: Thermal-induced, plastic deformation-induced, and thermomechanical-induced mechanisms. The thermomechanical-deformation-induced mechanism is the most likely;
2. Four main types of rail defect detection methods can be used to achieve wheel burn detection. They are electromagnetic, acoustic, visual, and axle box acceleration detection methods. Among them, magnetic flux leakage testing, magnetic Barkhausen noise testing, and eddy current testing can identify wheel burn at an early stage according to the different magnetic permeability and conductivity caused by the white etching layer. Acoustic emission testing takes advantage of the brittleness of the white etching layer to identify wheel burn at an early stage. Infrared thermography testing utilizes the difference in thermal diffusivity between WEL and the rail matrix to identify wheel burn at an early stage. When the WEL is developed, these methods can also detect near-surface cracks, but more research is needed for the fast detection and automatic identification of wheel burn. Automatic visual testing is not able to see through rails; white etching layer, surface cracks, spalling, indentation can be identified, but the depth of rail defects cannot be measured. Axle box acceleration measurement can detect severe wheel burn with plastic deformation and spalling developed from wheel burn.

Based on reviews and conclusions, a few future research directions of wheel burn are recommended below:

1. Improve the accuracy of individual inspection methods. Enhance the accuracy, repeatability, and speed of existing detection methods for wheel burn. For instance, research can focus on improving the sensitivity, resolution, and inspection speed of electromagnetic and visual testing techniques for wheel burn detection; research can also focus on improving the sensitivity of acoustic emission testing techniques for wheel burn monitoring.
2. Integrate multiple inspection methods. Combine different non-destructive testing techniques to enhance the accuracy of detecting various types of wheel burn. For example, integrating electromagnetic testing, visual testing, and axle box acceleration measurement can provide a more detailed assessment of wheel burn at different stages and can be mutually verified by each other. Acoustic emission testing can be used to monitor the long-term development of wheel burn.
3. Conduct theoretical research and experimental validation: Utilize finite element analysis and other methods to study the formation mechanism and crack extension of wheel burns. Further theoretical research and experimental validation are needed to gain a deeper understanding of these processes.
4. Conduct wheel burn identification and classification research: Utilize statistical approaches and neural network methods to achieve automatic identification and classification of wheel burn. Multi-source data fusion analysis is worth studying to increase the detectability rate of wheel burn.
5. Address challenges in implementing NDT techniques on railways: Overcome challenges associated with implementing NDT techniques in the railway industry, such as the requirement for specialized equipment and trained personnel. Collaboration among researchers, industry professionals, and regulatory bodies is necessary to develop standardized procedures for NDT testing on railways.

## Data Availability

Not applicable.
